# Is the patient aware of the difference between resurfaced and nonresurfaced patella after bilateral total knee arthroplasty? A systematic review of simultaneous bilateral randomized trials

**DOI:** 10.1186/s43019-022-00133-7

**Published:** 2022-02-14

**Authors:** Keun Young Choi, Yong In, Man Soo Kim, Sueen Sohn, In Jun Koh

**Affiliations:** 1 Joint Replacement Center, Eunpyeong St. Mary’s Hospital, 1021, Tongil-ro, Eunpyeong-gu, Seoul, 03312 Republic of Korea; 2 Department of Orthopaedic Surgery, Seoul St. Mary’s Hospital, Seoul, 06591 Republic of Korea; 3 Department of Orthopaedic Surgery, Inje University Sanggye Paik Hospital, College of Medicine, Inje University, Seoul, Republic of Korea; 4 Department of Orthopaedic Surgery, College of Medicine, The Catholic University of Korea, Seoul, 06591 Republic of Korea

**Keywords:** Patella, Patellar resurfacing, Bilateral, Total knee arthroplasty

## Abstract

**Purpose:**

The optimal practice of patellar management in total knee arthroplasty (TKA) remains controversial. This systematic review was conducted to compare patella-related (1) patient-reported outcome measures (PROMs), (2) clinical outcomes, and (3) reoperation rates after TKA with patellar resurfacing (PR) and nonresurfacing (NPR) in single patients undergoing bilateral patellar procedures during simultaneous bilateral TKA.

**Methods:**

This review included prospective bilateral randomized trials investigating patella-related PROMs, clinical outcomes, and reoperation (secondary resurfacing and patellar component revision) and other patella-related complications in single patients undergoing randomly assigned PR and NPR during bilateral TKA.

**Results:**

Six studies were included. There was no difference in PROMs between PR and NPR in five studies, whereas PR was found to be superior to NPR in one study. Five studies reported similar functional outcomes and complication rates between PR and NPR, while one study found better clinical outcomes and a lower complication rate in PR. Between-group secondary resurfacing and patellar revision rates were similar in all studies.

**Conclusions:**

The majority of patients who underwent bilateral patellar procedures could not tell the difference between PR and NPR following bilateral TKA. There were no differences in clinical outcomes or reoperation and complication rates between PR and NPR. No evidence was found to support routine PR.

Level of evidence: Therapeutic Level 1

## Introduction

Given the lack of solid evidence for a relationship between postoperative anterior knee pain (AKP) and patellar management in total knee arthroplasty (TKA), the optimal treatment of the patella in TKA has not been established [1–3]. Presently, there are three approaches to patella management during primary TKA: (1) always resurface, (2) never resurface, or (3) selectively resurface at the time of surgery [4]. While some studies have found that patellar resurfacing (PR) is beneficial in decreasing AKP [5, 6] and reoperation rates [7, 8], some surgeons choose to not perform PR because other studies show equivalent clinical results of PR and nonresurfacing (NPR) [9–14], or because the relationship between the intraoperative condition of the patellar cartilage and AKP after TKA with PR remains unclear [15, 16]. In addition, a substantial proportion of NPR patients have had secondary PR with no relief of AKP [15]. Moreover, PR is associated with its own patellofemoral complications that may lead to patellar component revision [17–23]. A selective approach to PR adopts the pros of both patellar management techniques [4], and until there is sound consensus on the optimal method of patellar management, surgeons will continue to practice based on their knowledge, training, and experience.

There are many randomized controlled trials (RCTs) that report a higher rate of AKP and reoperation in NPR compared with PR [7, 8, 24–28]. However, some studies assert that the higher risk of reoperation in NPR should be interpreted with caution because an inherent bias of easier indication for reoperation in NPR may artificially increase the reoperation rate [7, 29]. A side-by-side comparison between knees in single patients receiving different patellar treatments following bilateral TKA might be the most powerful and effective method for assessing differences in patient-reported outcome measures (PROMs) such as AKP, side preference, and satisfaction. There have been multiple RCTs comparing PR and NPR in TKA, but only a few studies have compared PR and NPR in single patients undergoing simultaneous bilateral TKA [6, 15, 30–33]. Whether or not patients are aware of the differences between PR and NPR knees, as reflected by PROMs and other results, remains to be determined [34].

Therefore, to answer the question of whether patients can tell the difference between PR and NPR in TKA, this comprehensive review was performed to compare patella-related PROMs, clinical and functional outcomes, and reoperation and patella-related complication rates between PR and NPR in single patients undergoing bilateral patellar procedures during bilateral TKA.

## Materials and methods

**Fig. 1:**
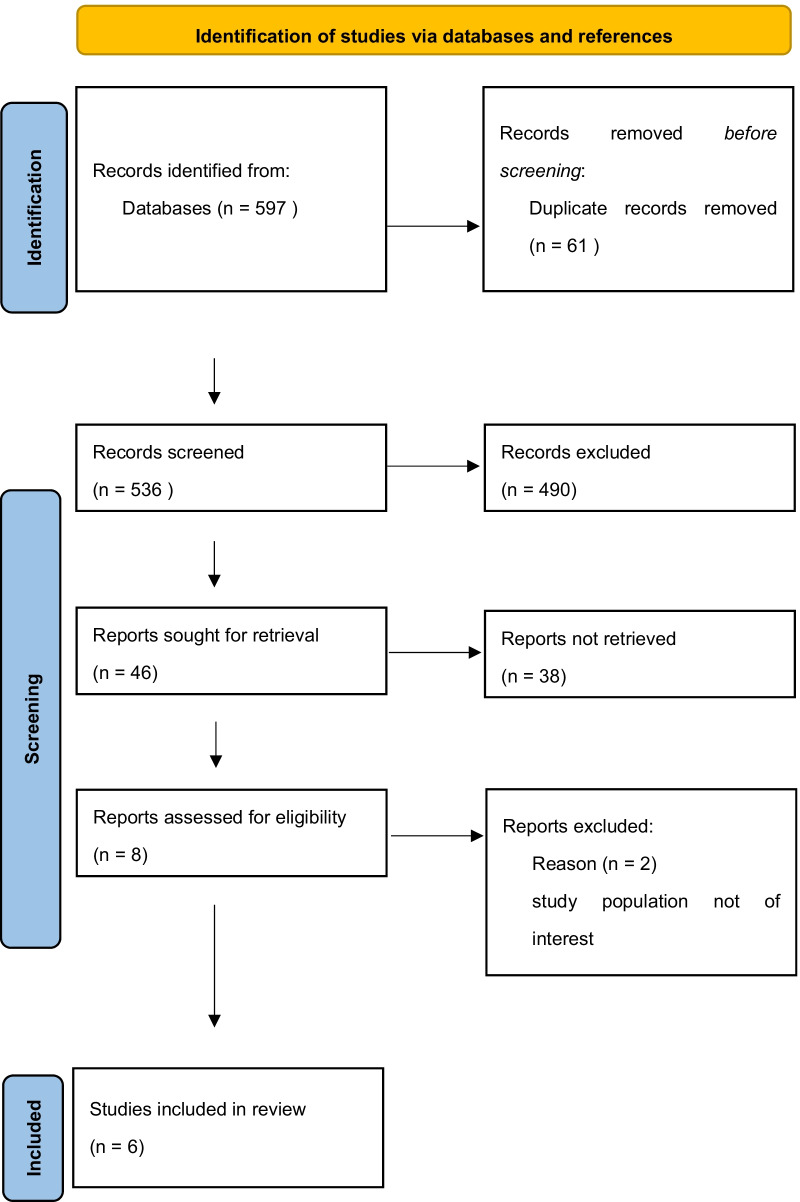
Flowchart of the search strategy

**Fig. 2:**
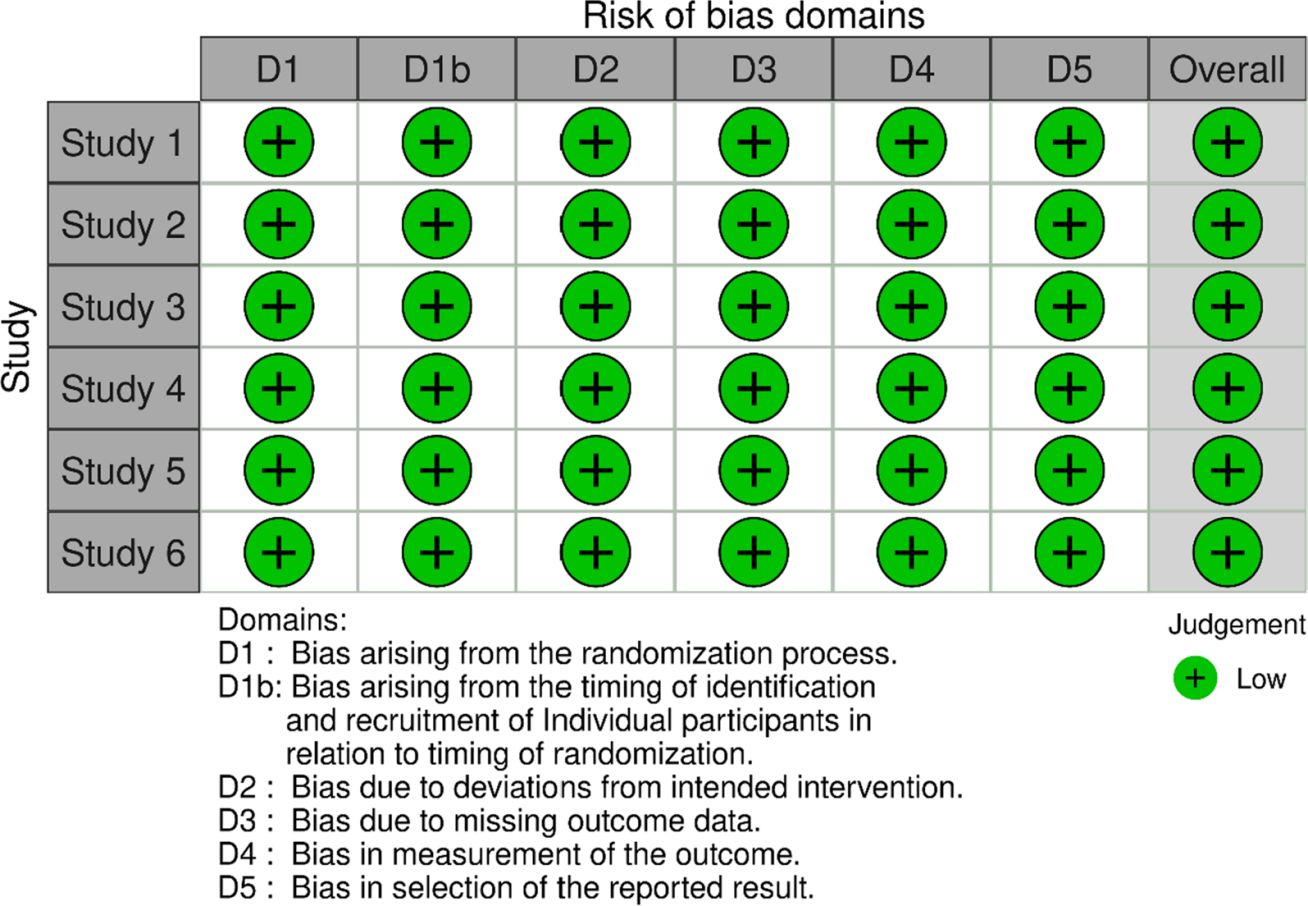
Risk of bias tool

This comprehensive review included only prospective RCTs comparing PR and NPR for patella-related PROMs, patellofemoral clinical and functional outcomes, and patella-related reoperation and complication rates in single patients undergoing bilateral patellar procedures during bilateral TKA. English language studies were identified by searching PubMed, MEDLINE, and EMBASE, and subsequently by searching the bibliographies of all relevant retrieved articles. The search included publications that (1) were publicly accessible on the internet, (2) were published in English after 1980, (3) presented bilateral TKA, (4) presented patella resurfacing techniques, (5) reported PROMs, and (6) reported objective data regarding clinical and functional outcomes between PR and NPR groups, as well as one of the following: perioperative radiologic findings including lower extremity axis, patella tilt, Insall–Salvati index, joint line position, residual patella bone after patella resurfacing, patella to groove distance, patella-related complications, revisional TKA, reoperation, preference, or satisfaction. The following terms were used for the initial literature search: ‘‘patella resurfacing’’ OR “patella retention” OR “simultaneous” OR “bilateral” OR ‘‘knee arthroplasty’’ OR ‘‘knee replacement’’ OR ‘‘total knee arthroplasty’’ OR ‘‘total knee replacement’’ OR ‘‘TKA’’ OR ‘‘TKR.’’ Two of the authors reviewed the full texts of all identified articles, and studies that did not report on any of the outcome variables listed above were excluded. The authors discussed any difference of opinion on study inclusion until they reached consensus. Of the 597 articles identified, 61 duplicates were removed and 490 were excluded because they did not meet the inclusion criteria. Thus, the detailed full content of 46 studies was reviewed, and 38 were excluded because the study populations did not receive simultaneous bilateral TKA (Fig. 1). Finally, six studies remained in this systematic review (Table 1), and those studies underwent quality evaluation using a risk of bias tool (Fig. 2). This study was exempted from the requirement for local Institutional Review Board approval because it is a retrospective systematic review of the literature.

**Table 1:** Summary of previous simultaneous bilateral randomized controlled trials (RCTs) comparing patellar resurfacing (PR) and nonresurfacing (NPR)

Author	Publication year	Journal	Country	Surgery period	Study design	Number of patients [age (Y)/F:M]	Follow up (Y)	Prosthesis name (company)	Preoperative PF arthritis
Burnett et al. [30]	2007	CORR	USA	1992–1993	Simultaneous bilateral RCT	20 (78/N/P)	10	CR Miller-Galante II (Zimmer)	No difference
Enis et al. [6]	1990	CORR	US	1984–1986	Simultaneous bilateral RCT	25 (65/21:4)	3.3	Townley (DePuy)	No difference
Smith et al. [31]	2008	JBJS (Br)	Australia	1998–2002	Simultaneous bilateral RCT	16 (N/P/N/P)	N/P	Profix (Smith & Nephew)	No difference
Ha et al. [32]	2019	Int Orthop.	China	2011–2012	Simultaneous bilateral RCT	60 (65/22:38)	5	Scorpio NRG (Stryker)	No difference
Koh et al. [15]	2019	KSSTA	South Korea	2012–2013	Simultaneous bilateral RCT	49 (70/48:1)	5	Lospa PS (Corentec)	No difference
Dong et al. [33])	2018	JOA	China	2013–2015	Simultaneous bilateral RCT	53 (68/30:23)	2.8	Genesis II PS (Smith & Nephew)	No difference

*USA* United States, *N/P* not presented, *F* female, *M* male, *Y* years, *CORR* Clinical Orthopaedics and Related Research, *JBJS* Journal of Bone and Joint Surgery, *Br* British, *Singapore Med J.* Singapore Medical Journal, *KSSTA* Knee Surgery, Sports Traumatology, Arthroscopy, *JOA* Journal of Arthroplasty

## Results

### Patient-reported outcome measures (Table 2)

**Table 2:** Summary of previous trials comparing patient-reported outcome measures between patellar resurfacing (PR) and nonresurfacing (NPR)

Author	Anterior knee	Global knee pain	Side preference	Satisfaction
Burnett et al. [30]	No difference	No difference	PR 37%/same 41%/NPR 22%	No difference
Enis et al. [6]	N/P	N/P	PR 45%/same 40%/NPR 15%	No difference
Smith et al. [31]	AKP in 31% (PR = NPR in 25%, NPR > PR in 6%)	No difference	N/P	No difference
Ha et al. [32]	PR: NPR = 3 (5%): 14 (23%) (*P* < 0.001)	N/P	PR 47%/same 46%/NPR 7%	N/P
Koh et al. [15]	No difference	No difference	PR 47%/same 8%/NPR 45%	N/P
Dong et al. [33]	N/P	N/P	PR 27%/same 52%/NPR 21%	N/P

*PR* patellar resurfacing, *NPR* nonresurfacing, *N/P* not presented

1. Anterior knee pain.

Among the six studies, three reported no difference in AKP between PR and NPR [15, 30, 31], two studies did not present clear AKP results [6, 33], and one reported a higher prevalence of AKP in NPR [32].

2) Global knee pain.

Global knee pain visual analogue scale (VAS) pain scores at the last follow-up were not different between PR and NPR in three studies [15, 30, 31], while this data was not clearly presented in three studies [6, 32, 33].

3) Side preference.

No difference in side preference between PR and NPR was found in three studies [15, 30, 33], while a greater preference for the PR side was found in two studies [6, 32]. Side preference was not reported in one study [31]. Interestingly, one study that reported better AKP in the PR group found no difference in side preference preoperatively but shifted toward a higher preference for the PR side with follow-up time [32].

4) Patient satisfaction.

Patient satisfaction was not different between PR and NPR in three studies [6, 30, 31] and not presented clearly in the other three studies [15, 32, 33]. Authors used their own nonvalidated questionnaire [30], validated satisfaction score [31], or did not describe a definite tool [6].

5) Western Ontario and McMaster Universities osteoarthritis index (WOMAC) score and forgotten joint score (FJS)

Only one study assessed these measures, and found no differences in WOMAC score or FJS between PR and NPR [15]. Five studies did not assess FJS and WOMAC scores [6, 30–33].

### Clinical outcomes including patellar scores (Table 3)

**Table 3:** Summary of previous trials comparing clinical outcomes between patellar resurfacing (PR) and nonresurfacing (NPR)

Author	Range of motion	Feller’s patella score	American Knee Society score	Radiologic findings
Burnett et al. [30]	No difference	N/P	No difference	No difference (anatomic axis, Insall–Salvati index patellar tilt, and subluxation)
Enis et al. [6]	No difference	N/P	N/P	No difference (loosening, otherwise N/P)
Smith et al. [31]	N/P	N/P	No difference	N/P
Ha et al. [32]	N/P	PR > NPR (*P* < 0.001)	PR > NPR (*P* < 0.001)	No difference (Insall–Salvati index, patellar tilt, and subluxation)
Koh et al. [15]	No difference	No difference	No difference	No difference (patella-to-groove distance, superior–inferior position, Insall–Salvati ratio, patellar tilt, and patella displacement)
Dong et al. [33]	N/P	No difference	No difference	PR < NPR in Insall–Salvati index (*P* < 0.05) No difference in patella tilt

*PR* patellar resurfacing, *NPR* nonresurfacing, *N/P* not presented

1. Range of motion (ROM).

No difference in ROM between PR and NPR was found in three studies [6, 15, 30]. ROM comparison was not presented in the other three studies [31–33].

2) Knee Society score (KSS) and Feller score.

Four studies reported no difference in KSS between PR and NPR [15, 30, 31, 33]. One study that reported a greater side preference for PR knees also reported a better KSS in the PR group at annual follow-up [32]. The KSS was not presented clearly in the other study [6]. There was no difference in the Feller score between PR and NPR in two studies [15, 33], while one study documented a better Feller score in the PR group [32]. Three studies did not present Feller scores [6, 30, 31].

3) Radiologic evaluation.

Among the six studies, four reported no difference in radiologic findings (anatomic axis, Insall–salvati index, patella tilt, patella subluxation, patella displacement, patella-to-groove distance, or superior–inferior position) between PR and NPR [6, 15, 30, 32]. One study did not present these data clearly [31] and one reported a higher Insall–Salvati index in NPR without clinical correlation [33].

### Patella-related reoperation and complication rates (Table 4)

**Table 4:** Summary of previous trials comparing complications between patellar resurfacing (PR) and nonresurfacing (NPR) after total knee arthroplasty

Author	Patellar clunk and crepitus	Secondary resurfacing following NPR	Revision of following PR	Other complications
Burnett et al. [30]	N/P	7.4% of NPR : AKP scores have not improved despite resurfacing	3.5% of PR for aseptic loosening at 6.9 years	N/P
Enis et al. [6]	N/P	0 (0%)	0 (0%)	N/P
Smith et al. [31]	N/P	0 (0%)	0 (0%)	N/P
Ha et al. [32]	10% of PR < 40% of NPR (*P* < 0.001)	0 (0%)	0 (0%)	0 (0%)
Koh et al. [15]	No difference	0 (0%)	0 (0%)	N/P
Dong et al. [33]	N/P	2% of NPR for severe AKP and subluxation	0 (0%)	N/P

*PR* patellar resurfacing, *NPR* nonresurfacing, *N/P* not present, *AKP* anterior knee pain

1. Secondary resurfacing following nonresurfacing and patellar component revision following resurfacing.

There was no secondary resurfacing following NPR in four studies [6, 15, 31, 32]. In one study, 7.4% of NPR knees underwent secondary resurfacing because of AKP, but AKP scores were not improved despite secondary resurfacing [30]. In the one remaining study, 1.9% of NPR patients required revision surgery to address severe AKP and patellar subluxation [33]. Among the six studies, five reported no revision of the patella component following PR [6, 15, 31–33] and the other study reported revision of resurfaced patella [30]. In this study, a patient with PR (3.5% of PR knees) had revision of resurfaced patella for aseptic loosening at 6.9 years, and the patient was unsatisfied with the outcome of TKA at the 10-year follow-up [30].

2) Patellar clunk

Among six studies, one reported no difference in rates of patellar clunk between PR and NPR [15] and one reported a higher incidence of patellar clunk in the NPR group [32]. Patellar clunk data were not presented clearly in the remaining four studies [6, 30, 31, 33].

## Discussion

The lack of understanding of post-TKA AKP makes it difficult to draw conclusions on the actual benefit of PR following TKA. Numerous studies have reported contradictory results regarding the relationship between PR and postoperative AKP [4, 7, 26, 35]. The optimal patellar management following TKA remains controversial, and the necessity of PR is a continuing subject of debate. Comparing outcomes in single patients undergoing different patellar procedures that are randomly assigned to each side during bilateral TKA can be one of the most powerful and effective strategies for determining whether patients can differentiate between PR and NPR. We conducted this literature review to determine whether PROMs, clinical outcomes, and reoperation and complication rates would be similar between PR and NPR knees in single patients undergoing simultaneous randomized bilateral patellar procedures during bilateral TKA.

The findings of this review suggest that patients who underwent bilateral TKA with different patellar management on each side were not aware of the differences between RP and NPR knees. Except in one Chinese study [32], which reported better AKP results in, and preference for, PR knees, there were no differences in PROMs in terms of AKP, global knee pain, preference, satisfaction, or WOMAC and FJS. This concurs with several meta-analyses reporting no difference in AKP between PR and NPR groups [24, 25], but contradicts several other meta-analyses reporting higher incidences of postoperative AKP in NPR [24–26, 28]. Post-TKA patellofemoral PROMs, including post-TKA AKP, are known to be affected by both patient and nonpatient factors including prosthesis design, surgical technique, degree of patellar chondromalacia, severity of preoperative AKP, and patellar tracking alteration [36]. By relying on a powerful matched pair design including only studies investigating bilateral patellar procedures in single patients, we can eliminate the bias of factors other than patella management that affect PROMs. The results of our study, together with previous studies, suggest that, in terms of PROMs, when all other factors are matched between groups, patients are not strongly aware of PR following TKA.

These findings also indicate that objective clinical outcomes were similar in PR and NPR. Most of the studies reporting on objective outcome variables including ROM, KSS, Feller score, out of chair and climbing stairs, and radiologic findings did not show significant differences between PR and NPR. Only one study, from China, reported better KSS and Feller scores in PR compared with NPR [32], while the other Chinese study reported a higher Insall–Salvati index in NPR [33] without any subjective or objective clinical difference. Again, these findings are in agreement with several previous meta-analyses reporting no difference in knee scores between groups in RCTs [24, 26]. Except for one Chinese study using implants with more anterior rather than posterior-stabilized prostheses [29], the findings of this systematic review, together with those of previous studies, indicate that post-TKA patellofemoral clinical and functional outcomes are not affected by the choice of patellar treatment.

This review found that complication rates and patella-related reoperation rates, including patellar component revision in PR and secondary resurfacing in NPR, were similar, and four of six studies reported no patellar component revisions or secondary resurfacing procedures [6, 15, 31, 32]. These findings are contradictory to several previous meta-analyses of RCTs that have suggested a higher risk of reoperation in NPR knees [8, 25–28]. However, our findings provide clues that reoperation rates in NPR groups may be artificially inflated by the option of secondary PR in NPR knees with persistent post-TKA AKP [7, 29]. By considering only RCTs investigating bilateral patellar procedures in single patients, we should have eliminated the inherent bias of easier indication to reoperation when the patella is not resurfaced. Furthermore, several previous studies have found that AKP persists in a substantial proportion of NPR patients who remain dissatisfied even after secondary resurfacing [37–39]. The results of this study, together with those of previous studies, indicate that postoperative AKP is not strongly associated with NPR, and the higher risk of reoperation after NPR should be interpreted with caution given that secondary resurfacing is the only remedial surgical option for postoperative AKP in NPR.

The findings of this study must be interpreted with the following limitations in mind. First, as we only performed an extensive search of the literature, we could not identify statistical significance or a concrete consensus. In addition, heterogeneities among studies regarding implant design; surgical techniques, including that of patella management in the NPR patient; surgical approach; pain management and rehabilitation protocols; and outcome variables make it difficult to judge the difference between groups. Additional studies with sufficient power investigating these issues in a more homogeneous fashion are required. Second, this systematic review focused on PROMs and clinical outcomes between PR and NPR, and we could not provide any information on benefits of NPR such as decreased operation time and improved cost effectiveness. Third, most of the studies that were included in this systematic review comprised relatively small numbers of patients (16–60) and were underpowered and susceptible to type-II error with respect to detection of all relevant outcomes. Finally, as the follow-up periods of the enrolled studies were relatively short (2.8–10 years), it is difficult to draw any conclusion on the long-term results. Despite these limitations, we believe that this study provides valuable information on the usefulness of patellar resurfacing in TKA.

## Conclusion

Current evidence shows that a broad majority of patients are generally unaware of any differences related to patellar resurfacing. In addition, functional improvement and reoperation rates between PR and NPR are not different. There was no evidence to support routine resurfacing of the patella in TKA. However, additional randomized trials with identical surgical techniques and prostheses, and with sufficient power would be necessary to ascertain the degree to which patellar resurfacing affects the patient experience after TKA.

## Data Availability

All data generated or analyzed during this study are included in this published article.
